# Intrinsically photosensitive retinal ganglion cells and visual processing: ipRGCs beyond non-image-forming functions

**DOI:** 10.3389/fnins.2025.1635101

**Published:** 2025-08-05

**Authors:** Hsing-Hao Lee

**Affiliations:** Department of Psychology, New York University, New York, NY, United States

**Keywords:** intrinsically photosensitive retinal ganglion cells (ipRGCs), blue light, visual processing, non-image-forming functions, melanopsin

## Abstract

Intrinsically photosensitive retinal ganglion cells (ipRGCs) are relatively newly discovered photoreceptors other than rods and cones. For the last decade, people have been considered ipRGCs to be primarily in charge of non-image-forming and cognitive functions. However, an increasing body of evidence has pointed out that ipRGCs also play a role in visual processing, such as contrast, brightness and color perception. In this mini-review, I listed what the caveats about those studies discussing how ipRGCs affect cognitive functions and how ipRGCs serve as image-forming functions under well-controlled condition.

## Introduction

Intrinsically photosensitive retinal ganglion cells (ipRGCs) are relatively newly discovered photoreceptors other than rods and cones. They are characterized by the photopigment—melanopsin, which makes them sensitive to light wavelength around 480 nm (Allen and Baño-Otálora, 2022; Do and Yau, 2010; Pickard and Sollars, 2011; Provencio et al., 1998; Provencio et al., 2000). Over the last decade, abundant of studies have proposed how ipRGCs serve as a “non-image-forming” photoreceptor and do not involve in visual functions (see Mahoney and Schmidt, 2024; Meng et al., 2025 for review). However, recently, increasing evidence obtained under better controlled light conditions suggests that ipRGCs contribute to human visual perception as well.

Blue light, which ipRGCs are most sensitive to, has been indicated to delay circadian rhythm (Chang et al., 2015; Daneault et al., 2016; Vandewalle et al., 2006), increase alertness (Beaven and Ekström, 2013; Phipps-Nelson et al., 2009; Souman et al., 2018), improve working memory (Suzuki et al., 2025; Vandewalle et al., 2013; Vandewalle et al., 2007; Vandewalle et al., 2009), improve task switching ability (Ferlazzo et al., 2014; but see Lee et al., 2021 showing no impacts), enhance creativity (Abdullah et al., 2016), expand time perception (Yang et al., 2018) and other cognitive functions (see Mahoney and Schmidt, 2024; Meng et al., 2025 for review). However, effects from blue light do not necessarily indicate that they originate from ipRGCs, especially when studies examining cognitive functions did not properly control luminance, background colors, and cone activation levels (Lucas et al., 2014; Mahoney and Schmidt, 2024; Yang et al., 2023). Specifically, most studies used different background light types (e.g., green or orange light, Chen and Yeh, 2019; Lee and Yeh, 2021) while failing to control luminance and the stimulation levels of cones and ipRGCs (Yang et al., 2023). This can possibly be a reason for mixed findings in several studies. A better way to control luminance would be using flicker photometry so that when the target color (i.e., blue) and the control color (e.g., green or orange) alternate at a frequency of around 10–20 Hz, the parvocellular pathway (P-pathway), which is primarily responsible for colors and sustained spatial response, will be suppressed (silenced). In the meantime, the magnocellular pathway (M-pathway) is in charge of the luminance and brightness for the two light sources, minimizing the perception of flicker and leads to identical luminance of the target and control colors (Bone and Landrum, 2004; Lee et al., 1988).

The techniques for controlling the background lights have significantly improved recently: By using silent substitution to create a metamer of the control light and stimulating ipRGCs with high versus low energy levels, researchers were able to dissociate the effects of cones and ipRGCs by using four-primary (e.g., Chien et al., 2020; Chien et al., 2023; Horiguchi et al., 2013; Tsujimura et al., 2010; Woelders et al., 2023; Yamakawa et al., 2019; Yang et al., 2023; Yang et al., 2018; Zele et al., 2018a) or five-primary lights (e.g., Cao et al., 2015; Uprety et al., 2022; Zele et al., 2018b), depending on the luminance level and if rods have saturated at the photopic level. Indeed, compared to studies examining the effects of ipRGCs on cognitive functions which have several caveats, studies examining the effects of ipRGCs on animal vision (e.g., Aranda and Schmidt, 2021; Barrionuevo and Cao, 2019; Hu et al., 2022; Patterson et al., 2020; Schmidt et al., 2011; Shi et al., 2025) and low-level human vision (e.g., Chien et al., 2023; Spitschan et al., 2017; Uprety et al., 2022) are under better controlled conditions.

ipRGCs contribute to color vision. More evidence has pointed out that ipRGCs are involved in color threshold (Barrionuevo and Cao, 2019) and color processing (Zele et al., 2018b), (see Barrionuevo et al., 2024) for review but Woelders et al. (2023) showing no effects of ipRGCs on color processing. If ipRGCs are changing human color vision, this could change how the trichromatic theory of color vision – the fundamental theory describing how three types of cones in the human retina can modulate the perception of a full range of colors – is mathematically computed, and the color matching functions should take this into account when examining the relations between cones and the spectral power distribution (SPD). Indeed, an experiment has revealed that traditional trichotomy can only explain perceptual sensitivity at the fovea but not in the periphery, because ipRGCs are primarily distributed in the periphery, thereby modulating the visibility there which was not account by the traditional trichotomy (Horiguchi et al., 2013). Future studies should carefully examine trichromatic theory of color vision at different eccentricities while taking ipRGCs’ distribution into consideration.

In addition to color vision, ipRGCs also contribute to contrast (Chien et al., 2023; Schmidt et al., 2014; Zele et al., 2019) and brightness perception (Besenecker et al., 2016; Brown et al., 2012; Cao et al., 2018; DeLawyer et al., 2020; Joyce et al., 2022; Lucas et al., 2020; Yamakawa et al., 2019; Zele et al., 2018b), but see Vincent et al. (2021) showing that ipRGCs do not impact luminance detection. For example, two blind patients without outer retina could report brightness percept and had pupillary light response when given short-wavelength light stimuli (Zaidi et al., 2007), indicating that rods and cones are not the only determining factors mediating visual processing. Indeed, Nugent and Zele (2024) have shown that ipRGCs alone can produce visual percept in response to both spatial and temporal patterns. Additionally, by using silent substitution, Chien et al. (2023) have shown that increased ipRGCs stimulation led to a higher contrast sensitivity at low spatial frequencies, and this effect varied across eccentricities according to the distribution of ipRGCs on the retina. Animal models also provided supporting evidence to this finding given that mice lacking melanopsin showed deficits in contrast sensitivity (Schmidt et al., 2014). Recently, Shi et al. (2025) also demonstrated that ipRGC activation can enhance the orientation selectivity in the primary visual cortex of mice by increasing preferred-orientation responses and narrowing tuning bandwidth.

Why do ipRGCs play a role in contrast, brightness, and color perception? The visual pathways mediated by ipRGCs are still perplexed (Joyce et al., 2022). Despite this, mice lacking rods and cones could still do the light detection task through melanopsin (Ecker et al., 2010). Animal models have shown that ipRGCs project extensively to the superior colliculus (SC) and dorsal lateral geniculate nucleus (dLGN), both of which play significant roles in visual perception rather than solely mediating non-image-forming functions (Allen and Baño-Otálora, 2022; Ellis et al., 2016).

For human, superior colliculus can process light information bypassing the cortical pathways and plays a critical role in controlling human eye movements (Binns, 1999; Liu X. et al., 2022; Manger, 2020). Interestingly, in human studies, melanopsin can stimulate the human homolog of frontal eye fields (Hung et al., 2017), which also engage in eye movements planning (Grosbras et al., 2005; Paus, 1996; Rivaud et al., 1994) and endogenous attention (Fernández et al., 2023). The neuroimaging evidence pointed out that ipRGCs are sending signals to neural substrates which are directly or indirectly connected to visual processing (Hung et al., 2017). More neuroimaging and modeling work with good quality of control are needed to verify ipRGCs’ mechanisms between brain structures and how they are shaping human low-level vision.

Contrary to previous arguments that ipRGCs are primarily in charge of non-image-forming functions (e.g., Mahoney and Schmidt, 2024; Meng et al., 2025), I argued that a variety of studies examining the effects of ipRGCs on cognition have serious caveats in controlling the light conditions. For example, due to the sluggishness and the receptive field properties of ipRGCs (Procyk et al., 2015), when examining their effects on cognition, researchers should carefully control the background lights (Mahoney and Schmidt, 2024), stimuli location (Barrionuevo et al., 2024), environmental lights (Webster et al., 2007) and the adaptation time needed to stimulate ipRGCs (Procyk et al., 2015; Yang et al., 2023). In vision studies, these factors are usually well-controlled, and ipRGCs undoubtfully play a role in human visual processing, such as contrast, brightness, and color processing (Allen et al., 2019; Joyce et al., 2022; Lucas et al., 2020). It is unknown, however, whether and how much are people metacognitively aware of the effects of ipRGCs on their cognition and perception (Cheng et al., 2023). Future studies could also consider how ipRGCs affect mid-level visual tasks, such as crowding, texture segregation, object localization, given the closely relationship between low- and mid-level visual processing in humans (Anderson, 2020; Jennings and Martinovic, 2014; Jones et al., 1997). Additionally, understanding how animal models’ findings can be applied to humans could further clarify the evolutionary conservation of visual and non-visual pathways, revealing their functional relevance in human physiology and behaviors (Emanuel and Do, 2015; Liu A. L. et al., 2022; Tünçok et al., 2025). Most importantly, whether and how the image-forming and non-image-forming functions of ipRGCs interact could further unveil how human visual and cognitive functions integrated to support our daily lives.
